# Expression analysis of *MND1/GAJ*, *SPATA22, GAPDHS* and *ACR* genes in testicular biopsies from non-obstructive azoospermia (NOA) patients

**DOI:** 10.1186/1477-7827-11-42

**Published:** 2013-05-15

**Authors:** Andriy Dorosh, Olina Tepla, Eva Zatecka, Lukas Ded, Karel Koci, Jana Peknicova

**Affiliations:** 1 Laboratory of Reproductive Biology, Institute of Biotechnology AS CR,v. v. i., Videnska 1083, 142 20, Prague 4, Czech Republic; 2 ISCARE I.V.F. a. s., Jankovcova 1569, Prague 7, Czech Republic

**Keywords:** Non-obstructive azoospermia, Human testes, Biopsy, Spermatogenesis, Gene expression, ICSI

## Abstract

**Background:**

High-throughput studies provide a wide spectrum of genes for use as predictive markers during testicular sperm extraction (TESE) in combination with ICSI. In this work, we used the specimens from testicular biopsies of men with non-obstructive azoospermia who underwent TESE to investigate the expression of spermatogenesis-related genes *MND1, SPATA22, GAPDHS* and *ACR*.

**Methods:**

Testicular biopsy specimens were subdivided into three groups: hypospermatogenesis (HS); maturation arrest (MA); and Sertoli cell-only syndrome (SCO). The levels of expression of the spermatogenesis-related genes *MND1, SPATA22, GAPDHS* and *ACR* in the testes were compared among these three groups using the reverse transcription polymerase chain reaction (RT-PCR) technique.

**Results:**

Analysis of the expression of spermatogenic genes in human testes with abnormal spermatogenesis showed different expression patterns in patients from different groups. Fertilization rate for studied set of patients was 66% and pregnancy rate 29%. For HS group fertilization rate was 72% and pregnancy rate 32%, while for MA group fertilization and pregnancy rates were 54% and 26%, respectively. Fertilization rates in relation to the studied genes were uniformly around 70%, pregnancy rates for ACR and GAPDHS genes were surprisingly low at 6% and 8% correspondingly.

**Conclusions:**

Analysis of the expression of genes involved in spermatogenesis can be a fast additional test for the level of spermatogenesis in testicular samples.

## Background

Testicular tissue is composed of many cell types serving as spatio-temporal environment for the male germ cell development. It is the only place in the male organism where meiosis occurs. After meiotic division, gene expression continues in haploid cells until chromatin condensation to produce proteins necessary for the final stages of spermatogenesis [1]. Germ cells also employ mechanisms for mRNA storage and delayed translation after chromatin has already been packaged. The final products of spermiogenesis are highly differentiated sperm cells, which are transcriptionally inactive. The rate of cell proliferation in testicular tissue is higher than in other tissues due to continuous sperm production. All these facts make gene expression analysis of testicular tissues extremely important.

Changes in the complex process of spermatogenesis caused by genetic background or environmental factors can lead to male infertility. Infertile men with no sperm cells in the ejaculate can father a child with the help of assisted reproduction techniques using testicular sperm. Intracytoplasmic sperm injection (ICSI) can be successful in men with non-obstructive azoospermia, but it cannot help patients with Sertoli cell-only (SCO) syndrome. Genome-wide expression studies of large groups of patients were performed to analyse the general changes in global gene expression of patients with infertility phenotypes [2-5]. This led to the identification of gene clusters that were differentially expressed in patients with spermatogenesis defects [4]. High-throughput studies provide a wide spectrum of genes for use as markers in the combination of testicular sperm extraction (TESE) with ICSI. Forty-seven genes exhibiting differential testicular gene expression associated with male infertility were detected in mice and 19 in humans [6]. They included genes involved in DNA repair, glutathione metabolism, proteolysis, spermatogenesis and stress response. These findings enabled the identification of markers for specific stages of spermatogenesis and the presence of somatic cells, thus improving infertility diagnostics.

In this work, we used the specimens from testicular biopsies of infertile men who underwent TESE for the ICSI procedure and investigated the expression of spermatogenesis-related genes. The *GAPDH* gene is expressed in somatic testicular cells and spermatogonia. Two genes, *MND1/GAJ* and *SPATA22*, are expressed prior to meiotic division and their corresponding proteins are involved in meiotic progression during spermatogenesis. *GAPDHS* and *ACR* genes reach the highest expression levels in haploid spermatids and are important for the sperm function.

## Methods

### Patients

Testicular tissue samples were collected from patients treated for infertility in ISCARE I.V.F. a.s. A total of 47 biopsy samples were obtained from azoospermic men aged 27–63 years. All patients enrolled in the study underwent testicular biopsies within their treatment and gave their written informed consent with donating the used material for the purposes of this research project. The study was approved by the institutional review board at the Institute of Biotechnology.

### TESE procedure, sperm extraction and ICSI

Testicular sperm extraction (TESE) was performed as previously described [7]. Briefly, small pieces of testicular tissue were placed in a Petri dish in Flushing medium (Medicult, Copenhagen, Denmark) and cupped up using two sterile needles. The fragmented tissue was assessed for the presence of motile spermatozoa under the phase contrast microscope. The suspension of cells was cultivated for 24–48 hours before injection or freezing procedure. Prior to the sperm retrieval procedure, a small piece of testicular tissue was taken for histological examination.

TESE samples were divided into three groups: hypospermatogenesis (HS), maturation arrest (MA), and Sertoli cells only syndrome (SCO), with a histopathology score counting according to Holstein et al. [8]. The corresponding grades were 6–8 for HS, grades 3–5 for MA and grade 2 for the SCO group.

ICSI procedures were carried out according to Silber et al. [9]. The sperm cells were incubated in droplets of 5 μl of Flushing medium (Medicult) with 30% of human serum for 2 hours followed by injection into the oocyte. The fertilization rate was assessed approximately 18 h after the injection by the presence of two pronuclei and second polar body and was quantified as a percentage of fertilized mature oocytes. Clinical pregnancy was confirmed by observing the gestational sac or detecting foetal heart beats.

### RNA purification

RNA purification was performed with the same piece of testicular tissue that was used for the sperm extraction and subsequently cryopreserved. Testicular biopsies with the residual medium were thawed directly in RNAlater RNA Stabilization Reagent (Qiagen, Chatsworth CA) and samples were homogenized with the Precellys 24 tissue homogenizer (Bertin Technologies, France). Total RNA was purified from the tissue samples using the RNeasy lipid tissue mini kit (Qiagen, Chatsworth CA) according to the manufacturer’s instructions and stored at −70°C. The concentration and purity of the purified RNA was determined by UV spectrophotometer Helios α (Thermo Electron Corporation, Marietta, USA) and confirmed by agarose gel electrophoresis. Human total testicle RNA, 1 mg/ml (Ambion®, Life Technologies™, Carlsbad CA), was used as a positive control.

### RT-qPCR

Reverse transcription and subsequent RT-qPCR was performed as previously described [10]. Briefly, prior to reverse transcription, purified RNA was treated with RNAse-free DNAse 1 (Fermentas, Burlington, Canada) for 40 min. Template cDNA was synthesized from 1 μg of total testicular RNA using SuperScript® III Reverse Transcriptase (Invitrogen, Life Technologies, Carlsbad CA) or RevertAid™ Reverse Transcriptase (Fermentas, Burlington, Canada) with combination of random hexamer and poly(dT) primers (1:1) in a Touchgene Gradient Thermal Cycler (Techne, Burlington, USA). The qPCR conditions were: initial denaturation for 15 min, followed by 40 cycles of denaturation at 94°C for 20 sec, annealing at 60°C for 30 sec, and extension at 72°C for 30 sec. Following PCR reaction, the melting curve was constructed by increasing the temperature from 72 to 95°C to ensure that the correct product is amplified in the reaction. PCR was repeated three times in doublets for each gene, and the average Ct was used for further analysis. Gene-specific primers for *PPIA*, *GAPDHS* and *ACR* were designed using the advantages of Primer3 software and BLAST alignment of Primer-BLAST service from NCBI [11]. Three best primer pairs overlapping the intron sequence were ordered and after pretesting, the best of them was used for gene expression analysis. Due to the large amount of pseudogenes for *GAPDH* gene in human genome [12], neither of the three primer pairs ordered was suitable because of the dimer formations, and the primers used were as in Barber et al. [13]. Primers for *MND1* and *SPATA22* were as in Okada et al. [4]. The *PPIA* (peptidylprolyl isomerase A (cyclophilin A)) gene was used as a reference gene. Primer properties are summarized in Table 1.

**Table 1:** Primer sequences

**Gene**	**Accession no.**	**Forward primer sequence (5' - > 3')**	**Reverse primer sequence (5' - > 3')**	**PCR product size, bp**	**Reference**
*MND1*	NM_032117.3	GTTGATGATGGTATGGTTGACTGTG	CCCTCAGACAACTGAGATTCCAGA	125	5
*SPATA22*	NM_032598.4	TGGCGTGAACATGCACAGAA	TTCGAATAATATGGGCCAGGTGTAA	89	5
*GAPDHS*	NM_014364.4	AAGGGGCCCATGGCTGGCATC	GCATCGAAGATGGACGAGTGGGT	92	this MS
*ACR*	NM_001097.2	TTGCTAAAGATAACGCCACGTGTGA	ATTTTTGCCGACGAAGCAGTGAGC	230	this MS
*GAPDH*	NM_002046.4	GAAGGTGAAGGTCGGAGTCAAC	CAGAGTTAAAAGCAGCCCTGGT	71	11
*PPIA*	NM_021130.3	CCCACCGTGTTCTTCGACATT	GGACCCGTATGCTTTAGGATGA	275	this MS

### Statistical analysis

Experimental data were analysed using STATISTICA 6.0. and GraphPad Prism 5.04. The differences between the control and experimental groups in the relative gene expression were analysed by KW ANOVA, and post hoc analysis was performed by Dunn‘s test. The p value that was equal to or lower than 0.05 was considered to be significant and was indicated with red asterisk in the column.

## Results

A total of 47 testicular biopsies were analysed. As the specimens were primarily used for sperm retrieval, in 13 cases the level or purity of isolated RNA was not sufficient for further studies. In the remaining 34 samples, morphological examination diagnosed nine biopsies as Sertoli cell only (SCO, 26%), 12 as maturation arrest at spermatocyte stage (MA), 12 as hypospermatogenesis with few sperm cells present (HS) and one sample as obstructive azoospermia with normal spermatogenesis. A commercial total testicular mRNA was used as a positive control for the gene expression.

Table 2 summarizes individual characteristics of the *in vitro* fertilization process, numbers of fertilized oocytes, embryo transfers and the cycles as well as occurrence of clinical pregnancy. Fertilization rate for all studied samples was 63%, in particular, for HS subset - 72% and MA subset – 54%. Pregnancy rate rate was 29% for whole set of patients, from this 32% for HS group and 26% for MA group. For samples with positive expression of studied genes fertilization rate for *GAPDHS* positive subset was 66%, *ACR* - 71%, *SPATA22* – 68%, *MND1* – 70%, pregnancy rates were 8%, 6%, 18% and 36% respectively.

**Table 2:** Fertilization outcomes in individual patients

**Sample**	**Spermatogenesis**	**Oocytes**	**Fertilized**	**Ebbryos transferred**	**Number of cycles**	**Clinical pregnancy**
1	HS	4	4	2	1	No
2	HS	8	5	2	2	No
3	HS	9	8	3	1	Yes
4	HS	1	1	1	1	No
5	HS	5	3	1	1	No
6	HS	12	7	2	2	No
7	HS	5	2	1	1	No
8	HS	4	4	2	1	Yes(2)
9	HS	13	11	2	1	Yes(2)
10	HS	18	12	2	2	No
11	HS	3	3	2	1	Yes/AB
12	HS	10	6	2	1	Yes
	HS total	92	66	22	15	7
13	MA	14	13	2	1	No
14	MA	8	5	4	2	No
15	MA	11	3	2	2	No
16	MA	9	5	2	1	No
17	MA	8	2	2	1	yes
18	MA	5	2	2	1	No
19	MA	8	4	4	3	yes
20	MA	5	5	2	1	Yes(2)
21	MA	5	4	NA	NA	No
22	MA	2	1	1	1	No
23	MA	9	4	1	1	Yes
24	MA	8	2	1	1	Yes/AB
	MA total	92	50	23	15	6
25	SCO	-	-	-	-	-
26	SCO	-	-	-	-	-
27	SCO	-	-	-	-	-
28	SCO	-	-	-	-	-
29	SCO	-	-	-	-	-
30	SCO	-	-	-	-	-
31	SCO	-	-	-	-	-
32	SCO	-	-	-	-	-
33	SCO	-	-	-	-	-
34	OA	4	2	2	1	No

HS – hypospermatogenesis; MA – maturation arrest; SCO – Sertoli cell only; OA – obstructive azoospermia; AB- miscarriage; N/A- data not available.

**Figure 1:**
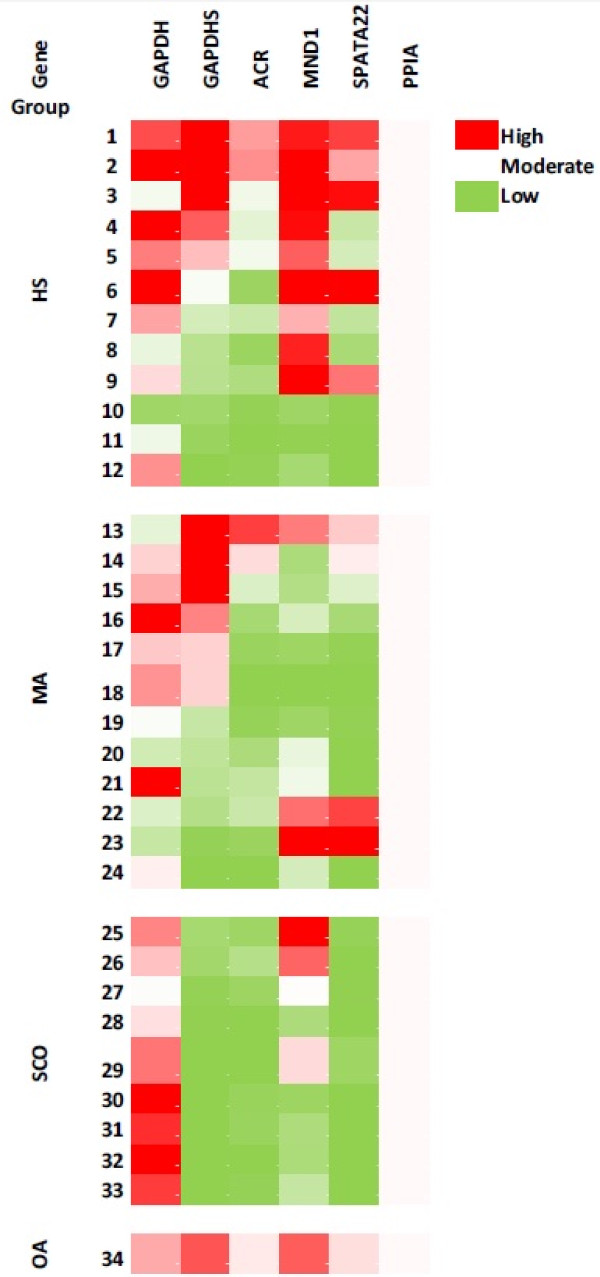
**Heat map representing the differential gene expression of the tested genes among individual samples and groups.** Red color indicates the highest gene expression (90th percentile), white color indicates moderated gene expression and green color indicates lowest gene expression (10% percentile). Other shades indicate intermediate values.

Testicular biopsy of OA showed a similar expression pattern to that of commercial testicular RNA (Figure 1). Three samples (10–12) from the HS group and six from the MA group (17–24) showed no or low expression of the studied genes. In the SCO group, two samples (25 and 26 in Table 2) showed decreased expression of the tested genes, whereas in the remaining seven biopsies only residual presence of GAPDHS, ACR and SPATA22 could be detected.

In patient 9 from the HS group and patients 22 and 23 from the MA group the expression of MND1 and SPATA22 was detected and no ACR or GAPDHS gene products were found.

**Figure 2:**
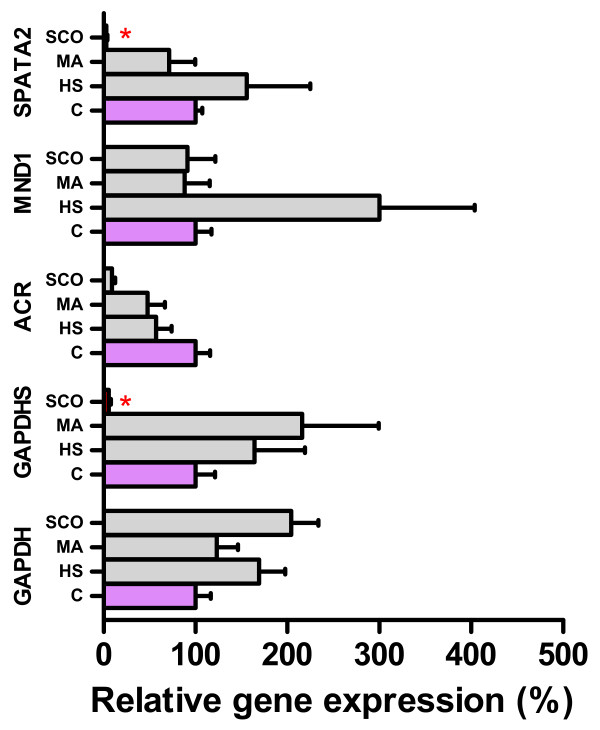
**The relative difference in the expression of tested genes among control and experimental groups of hypospermatogenesis (HS), maturation arrest (MA) and Sertoli cell- only syndrome (SCO).** Columns indicate the arithmetic mean of the relative expression of a particular gene, whiskers indicate SEM. Control group (C) is indicated by purple color and the relative expression was set as 100%. The statistically significant differences between control and experimental groups are indicated by red color of the column or red asterisk.

Next, we looked whether any difference in relative expression of the studied genes could be found between the histological groups of HS, MA and SCO. Relative gene expression was significantly decreased for *SPATA22* and *GAPDHS* in the SCO group (Figure 2). The *ACR* gene was downregulated as well, but due to high inter-individual differences and the low number of studied samples in the groups the decrease was not significant.

## Discussion

The main goal of all analytic procedures in patients with non-obstructive azoospermia is to quickly obtain reliable data for successful prediction of testicular sperm retrieval. Some laboratories attempted to predict spermatogenesis with non-invasive techniques with differing success [14-16]. To date, the only generally accepted reliable predictor of successful TESE is testicular histology [17]. Analysis of the germ cell-specific gene expression in testicular samples can provide an additional, supplementing approach to increase the prediction of positive TESE outcome.

Testicular transcriptome consists of gene expression patterns of both somatic and germ cells and has been intensively studied in recent years [3]. The first studies were focused on describing the global testicular gene expression and identifying testicular genes in mice [18] and human [19]. Shima et al. [1] took advantage of the first synchronous wave of spermatogenesis in pubertal mice to locate the gene products to specific testicular cells. In a different approach, germ cells were purified for high-throughput analysis of cell-specific gene expression studies on animal models [20,21]. The data from the above-mentioned studies provides a vast number of possible gene candidates as markers of spermatogenesis that fulfil the criteria of testis-specific gene expression, are transcribed and translated at specific time points of spermatogenesis, and their presence indicates correct gamete development. In addition, it was shown recently that besides changes in mRNA levels in azoospermic men, the miRNA expression is also altered [22].

Another approach to TESE sample analysis is to verify whether the expression of a single gene or a couple of genes can be used as a simple indicator of positive sperm retrieval in patients undergoing treatment in infertility clinics. Detection of DAZ (deleted in azoospermia), DAZL (DAZ-like) and protamine 2 (PRM2) mRNA in testicular samples was shown to be an informative tool for spermatogenesis evaluation [23]. Similar results were obtained with the *BOULE* mRNA occurrence [24]. Ando et al. showed that expression of *VASA*, *ODF1*, *ODF2* (outer dense fiber 1 and 2) and *SMCP* (sperm mitochondria-associated cysteine-rich protein) genes was significantly stronger in the successful TESE group [25]. Other genes expressed in post-meiotic stage may also be good candidates for the prediction of successful fertilization.

In our study, we followed expression of five genes that are expressed at certain stages of the spermatogenesis process and are important for meiosis and sperm development. The *MND1* and *SPATA22* genes were selected for spermatogenesis characterization in azoospermic patients because of the known significant gene expression differences in different non-obstructive azoospermic patients [4]. The Mnd1/Gaj protein plays an important role in homologous chromosome pairing and efficient cross-over during meiosis [26]. The *SPATA22* gene product was shown to be involved in meiotic progression of germ cells in mice [27]. The *ACR* mRNA appears first in pachytene spermatocytes, reaching the maximum levels in round spermatids, and preproacrosin protein appears in spermatids [28]. The reason for non-obstructive azoospermia in this case may be interrupted or incomplete spermiogenesis or sperm maturation. Nevertheless, the loss of acrosin protease activity does not lead to infertility in mice and spermatozoa from knock-out mice can penetrate zona pellucida of the oocyte [29]. The gene encoding sperm-specific glyceradehyde-3-phosphate dehydrogenase, *GAPDHS*, was shown to be expressed solely in haploid round and elongating spermatids [30,31] to replace the function of somatic *GAPDH* gene, whose expression ceases in germ cells. *GAPDHS* gene expression may be a good marker for spermatogenesis analysis, as its transcription and translation are temporarily separated and mRNA forms a complex with an RNA-binding protein, which results in translation and mRNA degradation delay [32]. Therefore, expression of the *GAPDHS* gene might be detectable even in poor-quality or low-quantity testicular samples. Poor detection of gene expression in nine biopsies (10–12 from HS and 17–24 from MA groups) suggests that in the tissue analysed for RNA purification, spermatogenesis was either greatly reduced or RNA was probably purified mainly from somatic cells. An interesting pattern of gene expression was observed in patients 9, 22 and 23 with normal expression of *MND1* and *SPATA22* genes and residual levels of *GAPDHS* and *ACR* genes. This might indicate that spermatogenesis in these patients continues undisturbed until meiosis, but either meiosis or spermiogenesis is somehow impaired.

Fertilization and pregnancy rates in population of studied patients were in accordance to those from previous studies. Fertilization rates for subsets of samples with positive expression of studied genes showed uniform fertilization rates around 70%, only *MND1* gene was expressed in samples from SCO group where sperm cells could not be retrieved. Surprisingly, for most promising markers of final steps of spermatogenesis, *ACR* and *GAPDHS*, pregnancy rate was below 10%. This indicates that expression analysis of present testicular genes cannot indicate successful pregnancy in studied couples. It is highly probable that in this process, oocyte and embryo quality have higher impact on the successful pregnancy. Moreover, low number of studied samples does not allow drawing any correlation between specific gene expression and fertilization outcome.

All four studied genes are expressed at different stages of spermatogenesis, and *ACR, SPATA22* and *GAPDHS* gene expression might be a good predictor of successful TESE outcome. Nevertheless, analysis of a greater number of testicular biopsies is needed to confirm that changes in gene expression of the selected genes can serve as markers to justify repeated TESE. Another thing to consider is that spermatogenesis is a dynamic process and TESE sample analysis provides information about the gene expression and spermatogenesis state at a single time point only.

A novel non-invasive approach to prediction of the state of spermatogenesis and pathophysiology of testicular tissues via the detection of germ cell-specific mRNA traces in seminal plasma was introduced recently [33,34]. Future analysis of germ cell-specific genes, including those from our study, or *GAPDH/GAPDHS* ratio in cell-free seminal plasma from azoospermic patients might become a promising non-invasive tool for TESE success prediction. The advantage of this technique is that the seminal analysis provides complex whole-testis physiology in comparison to the TESE sample representing a limited region of the analysed tissue.

To sum up, non-obstructive azoospermia is a complex pathophysiological state that leads to changes of gene expression in the testes, and understanding this process may lead to identification of the molecular markers of the spermatogenesis level.

## Conclusions

Expression analysis of genes whose expression occurs exclusively in germ cells during spermatogenesis provides sensitive confirmation of the histological diagnosis of SCO syndrome, as it was decreased in all histologically identified SCO patients. In the case of maturation arrest or hypospermatogenesis, gene expression analysis could help determine the stage at which spermatogenesis arrest occurs and be a key factor in making the decision whether repeated TESE could be considered after previous ICSI failure.

## Competing interests

The authors declare that they have no competing interests.

## Authors’ contributions

AD carried out the cDNA synthesis, qPCR analysis, participated in the study design and drafted the manuscript; OT and KK collected the samples and corrected the manuscript; EZ performed RNA work and critically read the manuscript; LD participated in the design of the study and performed the statistical analysis; JP participated in the design and coordination of the study and helped to draft the manuscript. All authors read and approved the final manuscript.
